# Evaluating the Multifunctional Performance of Structural Composites for Thermal Energy Storage

**DOI:** 10.3390/polym13183108

**Published:** 2021-09-15

**Authors:** Giulia Fredi, Andrea Dorigato, Luca Fambri, Alessandro Pegoretti

**Affiliations:** INSTM Research Unit, Department of Industrial Engineering, University of Trento, Via Sommarive 9, 38123 Trento, Italy; andrea.dorigato@unitn.it (A.D.); luca.fambri@unitn.it (L.F.); alessandro.pegoretti@unitn.it (A.P.)

**Keywords:** multifunctional composites, thermal energy storage, phase change materials, thermal properties, mechanical properties, multifunctional efficiency

## Abstract

The simultaneous need for high specific mechanical properties and thermal energy storage (TES) function, present in several applications (e.g., electric vehicles), can be effectively addressed by multifunctional polymer-matrix composites containing a reinforcing agent and a phase change material (PCM). The PCMs generally decrease the mechanical properties of the host structural composites, but a multifunctional composite can still be beneficial in terms of mass saving, compared to two monofunctional units performing the structural and heat management functions individually. To quantify any possible advantages, this paper proposes an approach that determines the conditions for an effective mass saving at the system level and ranks the investigated structural TES composites with a parameter called multifunctional efficiency. It is found that the potential mass saving is higher when the volume fraction of the reinforcement is kept constant also when the PCM fraction increases or when the single phases (reinforcement, PCM) are themselves multifunctional.

## 1. Introduction

Thermal energy storage (TES) is a key technology for more efficient and rational use of energy resources, as it allows the temporary conservation of excess heat that can be released at another time or place, as the demand for thermal energy overcomes its availability [1,2]. Among the most promising materials for TES in the low-medium temperature range (0–100 °C) are the organic solid–liquid phase change materials (PCMs), such as paraffin waxes, poly(ethylene glycol)s (PEGs), fatty acids, and fatty alcohols, which accumulate heat when they melt and release it upon crystallization [3,4,5]. PCMs can store a high amount of latent heat at a nearly constant temperature, and thus they are often used to maintain the temperature in a specific range [6]. This makes them suitable for thermal management applications, such as to regulate the indoor temperature in buildings, to avoid overheating of batteries and other electronic devices, or to produce smart thermoregulating textiles [7,8,9,10,11,12,13]. To prevent leakage and loss of material above the melting temperature, PCMs can be either encapsulated in macro-, micro-, or nano-shells or “shape-stabilized” in porous materials or nanofiller networks [14,15,16,17,18,19].

TES systems generally represent a supplementary component that is added to the main structure of a device, which can increase the volume and weight of the device itself. However, in applications where weight and volume savings are critical design parameters, it would be beneficial to embed heat storage/management functionalities directly into structural or semi-structural materials. In this context, polymer-matrix composites have the potential to be designed as multifunctional materials with both structural and non-structural functions [20,21,22,23]. They gather in one material the properties of two or more phases, and their composition can be fine-tuned to obtain the desired combination of properties [24,25,26]. Lightweight polymer composites combining good mechanical properties and TES capability could be applied in the automotive industry, where the diffusion of lightweight structures could complicate the thermal management of the environment in the cockpit, or in the portable electronics field, where the reduction in volumes and masses also limits the space available for the cooling system. The scientific literature reports some examples of polymers containing a PCM, such as 3D printable thermoplastic polyurethane containing paraffin microcapsules, polypropylene-paraffin compounds, and polyurethane foams containing a microencapsulated PCM [13,27,28,29,30,31,32,33]. However, little has been carried out to deeply investigate their mechanical properties and enhance them with a reinforcing agent. In fact, most of the reported PCM-containing polymers are not suitable for load-bearing applications.

There are very few examples of reinforced polymer composites containing a PCM. For example, Yoo et al. [34,35,36,37] prepared and characterized an epoxy/glass laminate containing paraffin microcapsules. More recently, our research group has developed many examples of reinforced polymer composites containing a PCM, such as carbon/epoxy or glass/epoxy laminates containing microencapsulated or carbon-nanotubes (CNT)-stabilized paraffin [38,39,40,41], polyamide-based composites containing continuous or discontinuous fibers and microencapsulated or shape-stabilized PCMs [40,42,43,44,45], discontinuous carbon fibers/epoxy composites with paraffin microcapsules [46], carbon fiber laminates with a microencapsulated PCM and a reactive acrylic thermoplastic matrix [47,48], epoxy/hollow-glass-microspheres syntactic foams containing paraffin microcapsules [49], and biodegradable wood/thermoplastic starch laminates containing PEG [50]. The results of the characterization showed that the introduction of an organic PCM in a fiber-reinforced composite does increase its TES capability and thermal management properties, but it often impairs its mechanical performance, especially at high PCM weight fractions. This occurs because the addition of a third phase unavoidably decreases the maximum fiber volume fraction, and also because the commercial microencapsulated PCMs are generally not intended as fillers for polymer matrices, and therefore their thermomechanical and surface properties are not optimized for this purpose. Nevertheless, combining structural and TES functions in a composite material may still be advantageous in terms of mass and volume saving, compared to two monofunctional units performing the structural and TES functions individually [51]. To quantify this advantage, it is important to develop objective selection and design criteria that consider all the multiple and sometimes competing design requirements of such structural TES composites.

Hence, this work aims to develop a parameter to quantitatively evaluate the multifunctionality of composites that perform both the structural and TES functions. First, a multifunctional epoxy/carbon laminate containing paraffin microcapsules is presented and discussed as a case study, to clarify the potentialities and challenges of this design concept with a practical example. Then, a criterium is discussed to minimize the mass of a component with both thermal energy storage and structural functions, and a multifunctionality parameter is developed that quantifies the mass saving at the system level. This parameter is then applied to evaluate and rank the structural TES composites investigated by our group. This approach allows the development of objective design principles and material selection guidelines, fundamental to maximize the advantages of using a multifunctional material.

## 2. Case Study: Epoxy/Carbon Laminates Containing Paraffin Microcapsules

This paragraph reports a case study of unidirectional carbon/epoxy laminates with TES capability. It is important to stress that this example of a structural TES composite has already been published in a previous work of our group [41] with all the experimental details and results of the characterization, but it is reported here as a case study to illustrate with a practical example the concept of structural TES composite, its preparation, and thermomechanical characterization. The reader can refer to [41] for all details about the experimental part and the discussion of the results omitted in this work.

In these carbon/epoxy laminates, the TES function is fulfilled by a microencapsulated PCM, contained in various weight fractions (up to 22 wt% of the total laminate mass). The PCM consists of commercial core-shell microcapsules (MCs) with a paraffinic core and a melamine-formaldehyde-based shell, with a total declared average diameter of 15–30 μm, melting enthalpy of 200–210 J/g, and melting temperature of 43 °C.

Laminates were prepared by hand layup and vacuum bagging methods. First, the two epoxy (EP) components (base and hardener) were mixed together, and then the MCs were added at various weight concentrations, i.e., 0 wt%, 20 wt%, 30 wt%, or 40 wt% of the total matrix (EP + MCs) weight. The resulting mixtures were thoroughly stirred to homogenize the composition and used as matrices to prepare laminates via hand layup, with unidirectional carbon fiber (CF) fabric. All laminates were vacuum-bagged, cured at room temperature for 24 h, and post-cured at 100 °C for 10 h. Thin (8-ply, type A) and thick (16-ply, type B) laminates were prepared to obtain specimens suitable for a broad range of microstructural and thermomechanical characterization techniques. Samples will be from now on labeled as EP-MCx-CFu-A (thin) or EP-MCx-CFu-B (thick), where x represents the initial MC weight fraction in the matrix (x = 20, 30, 40). The full details on the materials and sample preparation are reported in our previous work [41].

**Figure 1:**
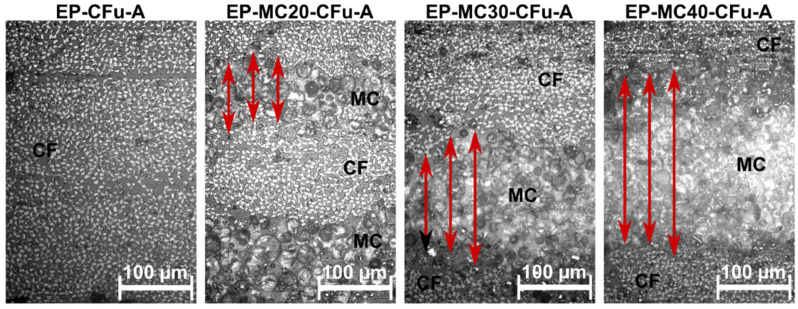
Optical microscope micrographs of the polished cross-section of the laminates EP-MCx-CFu-A. Red arrows indicate the thickness of the interlaminar region rich in microcapsules (MC) between two carbon fiber (CF) layers.

Figure 1 shows optical microscope (OM) micrographs of the type-A laminate cross-sections. The MCs are preferentially distributed in the interlaminar region, and the thickness of this MC-rich zone increases with the initial MC content, as indicated by red arrows in Figure 1. This phenomenon, accentuated by the difference in sizes of MCs (average diameter 20 µm) and CF (average diameter 7 µm), has two main consequences. First, the thickening of the interlaminar region increases the laminate thickness given the same number of laminae and decreases the fiber volume fraction. Second, the accumulation of a relatively weak phase in the interlaminar zone may generate a preferential path for damage propagation, thereby impairing the interlaminar strength.

**Figure 2:**
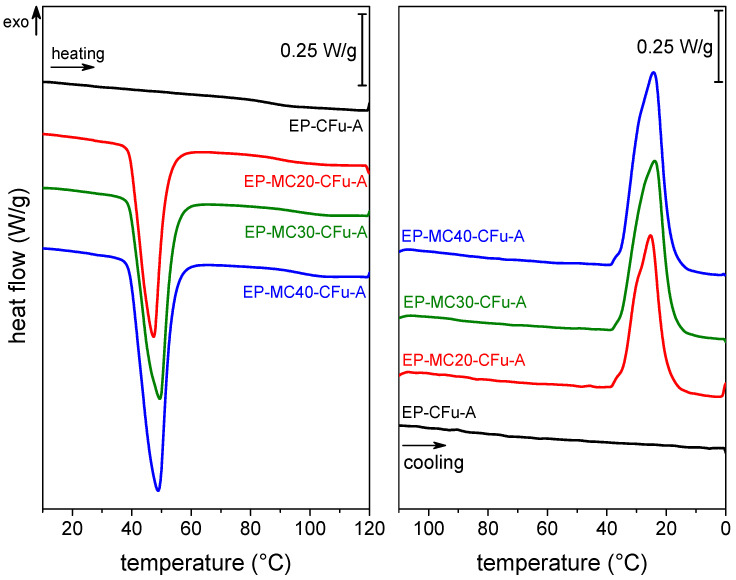
Representative DSC thermograms of laminates EP-MCx-CFu-A. First heating scan and cooling scan. Reprinted with permission from [41].

To estimate the effective mass fraction of MCs in the final composites, the phase change enthalpy of the PCM in the composites was measured with differential scanning calorimetry (DSC) and compared with that of neat microcapsules. The endo-/exothermic peaks related to the PCM phase change are evident in the DSC thermograms of the laminates Figure 2. The endothermic signal is visible at 40–60 °C in the heating scan and the exothermic signal at 40–15 °C in the cooling scan. The melting and crystallization enthalpy values $\Delta H_{m}\;$and $\Delta H_{c}\;$(Table 1) increase with the MC loading, up to 48.7 J/g for the sample EP-MC40-CFu-A, and this evidences that the TES properties increase with the PCM content. Of course, the weight fractions of MCs in the initial EP/MC mixtures are known (0, 20, 30, and 40 wt%), but that in the laminates is influenced by the fiber- and matrix weight fractions, which are in turn dependent on the matrix viscosity and thus on the initial MC loading. In fact, MCs considerably raise the viscosity of the EP/MC mixtures, and this hinders the flowing of the matrix out of the fiber fabric during vacuum bagging, thereby increasing the final matrix weight fraction.

The MC weight fraction in the laminates ($\omega_{MC},\;$Table 1) is calculated by dividing the $\Delta H_{m}\;$of the laminates by that of neat MC. The MC weight fraction ranges from 13.2 wt% to 22.0 wt% and increases with the initial MC loading. These values were combined with those of the weight fraction of CF and EP (measured through thermogravimetry) and those of density of each phase and used to determine the volumetric composition and the porosity of the laminates (Table 1). As expected from the optical micrographs (Figure 1), an increase in the MC concentration determines a decrease in the fiber volume fraction ($\vartheta_{f}$) and an increase in porosity ($\vartheta_{v}$), due to the increased matrix viscosity.

**Figure 3:**
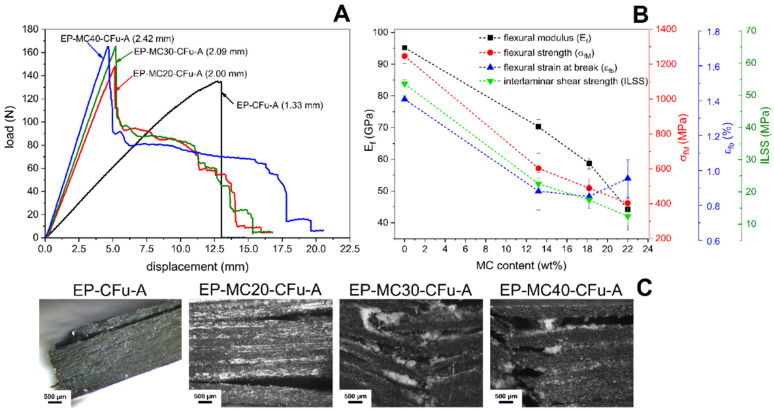
Results of the 3-point bending tests on the laminates EP-MCx-CFu-A and short-beam shear tests on the laminates EP-MCx-CFu-B. (**A**) Representative load–displacement curves. The thickness of the laminates is indicated; (**B**) Flexural modulus ($E_{f}$), flexural strength ($\sigma_{fM}$), flexural strain at break ($\varepsilon_{fb}$) and interlaminar shear strength (ILSS) as a function of the MC weight fraction; (**C**) Photographs of the specimens after 3-point bending tests. Reprinted with permission from [41].

The mechanical properties of these laminates were investigated through quasi-static tensile and 3-point bending tests, short-beam shear tests, and mode I interlaminar fracture toughness tests. The bending load–displacement curves of all laminates (Figure 3A) show an initial linear region, but for the neat EP-CFu laminate, the load drops to zero immediately after the maximum value, indicating a sudden and catastrophic failure, which was reported as a sign of good interlaminar adhesion [52,53]. On the other hand, MCs change the failure mode and make the load decreasing stepwise after the maximum, indicating energy dissipation also during damage propagation. In these cases, the failure starts frequently in the MC-rich interlaminar region, as observable from the photographs of post-test specimens (Figure 3C), and not in the tensile-stressed region. The decrease in the fiber volume fraction and the weaker interlaminar region are at the basis of the decrease in the flexural modulus ($E_{f}$), strength ($\sigma_{fM}$), strain at break ($\varepsilon_{fb}$), and interlaminar shear strength (ILSS) (Figure 3B).

**Figure 4:**
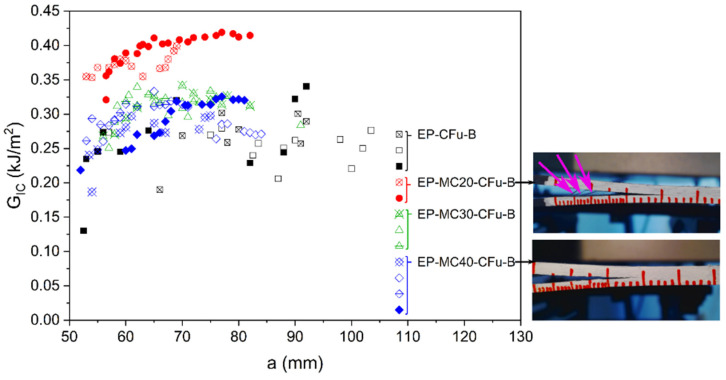
Results of the interlaminar fracture toughness tests. R-curves of the laminates EP-MCx-CFu-B and pictures of the EP-MC20-CFu-B specimens (arrows indicate fiber bridging) and EP-MC40-CFu-B (no evidence of fiber bridging). The results of two to four specimens are shown for each sample. Reprinted with permission from [41].

The interlaminar properties were studied more in detail through mode I interlaminar fracture toughness on double cantilever beam specimens, and the results allow the calculation of the R-curves (Figure 4) and the mode Ι critical strain energy release rate for crack initiation ($G_{Ii}$) and steady-state propagation ($G_{Ic}$) (Table 2). Both $G_{Ii}$ and $G_{Ic}$ increase with a small amount of MC, as they are higher for EP-MC20-CFu-B than for EP-CFu-B. However, $G_{Ii}$ and $G_{Ic}\;$decrease with a further increase in MC fraction, likely due to excessive thickening and weakening of the interlaminar zone, caused by poor MC/EP adhesion and poor mechanical properties of MC. The observation of the specimens during testing highlights that the neat laminate and the sample EP-MC20-CFu-B manifest fiber bridging (Figure 4), which is not observed on the laminates with a higher MC amount. Therefore, in the MC-richer laminates, the crack propagates within the matrix rather than at the fiber-matrix interface, while in the laminate EP-MC20-CFu-B fiber bridging may sum up with other possible MC-activated mechanisms, such as particle debonding, crack pinning, crack deflection, and micro-cracking.

The prepared laminates were also subjected to dynamic-mechanical thermal analysis (DMTA). This technique allows following the PCM phase transition by observing the effects of its melting and crystallization on the dynamic-mechanical parameters of the host composites. In this way, the phase transition can be studied not only from the point of view of the heat exchange, as in DSC, but also from a dynamic-mechanical approach, which is very powerful to analyze all the important properties of a multifunctional composite simultaneously.

**Figure 5:**
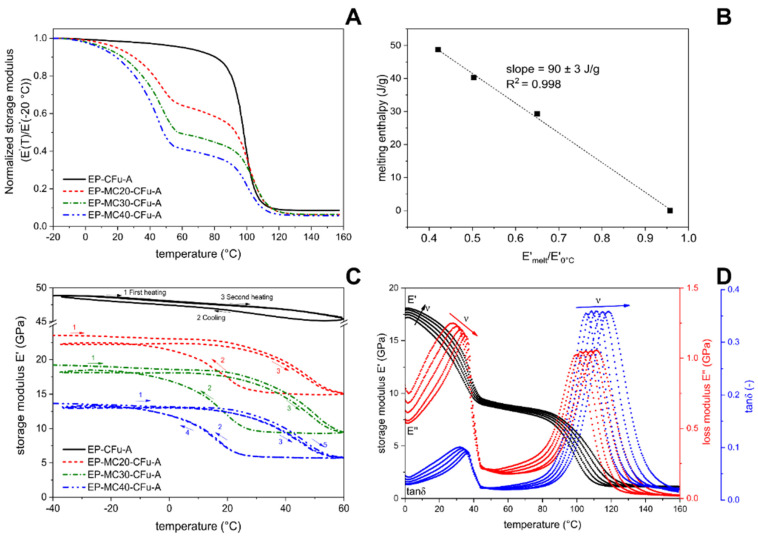
Results of single-cantilever DMTA tests on laminates EP-MCx-CFu-A. (**A**) Single-frequency tests (1 Hz). Storage modulus ($E^{\prime }$) normalized to the value at −20 °C; (**B**) Single-frequency tests. Value of $E^{\prime }$ after PCM melting (60 °C) normalized to the value at 0 °C, as a function of the melting enthalpy, with the results of the linear fitting; (**C**) Cyclic tests. Storage modulus on a heating–cooling–heating cycle; (**D**) Multi-frequency (0.3-1-3-10-30 Hz) test on the sample EP-MC40-CFu-A. Reprinted with permission from [41].

To fully explore the potentialities of this technique, DMTA was performed in three testing modes, i.e., single-frequency scans, heating-cooling cycles, and multifrequency tests. In single frequency scans (Figure 5A,B), the storage modulus ($E^{\prime }$) of the laminates containing MCs decreases markedly not only at the glass transition temperature of the epoxy matrix (~100 °C), but also at the melting of the PCM (~40 °C). The amplitude of this step increases linearly with the MC weight fraction, and the linear fitting of this correlation is surprisingly good (R^2^ = 0.998). This decreasing step is almost completely recovered during cooling. In fact, DMTA heating–cooling cycles (Figure 5C) show that the PCM crystallization is associated with an increasing $E^{\prime }$ step occurring with a certain hysteresis compared to the melting one, which is especially due to thermal inertia. The final value of $E^{\prime }$ after the first complete heating–cooling cycle is ~95% of the initial value, while further thermal cycles bring a negligible decrease in $E^{\prime }$. Since these structural TES laminates must withstand repeated heating–cooling cycles across the PCM melting temperature, this recovery of $E^{\prime }$ upon crystallization is very promising for future applications. This phenomenon could also be exploited to design composites with dynamically tunable stiffness.

Finally, multifrequency DMTA tests (Figure 5D) confirm the decreasing step in$\;E^{\prime }$ at the PCM melting, accompanied by evident peaks in $E^{\prime \prime }$ and tanδ signals. Moreover, these tests showed that a strong dependence on the applied frequency is observable not only at the glass transition but also at the PCM melting. In this temperature range, the frequency sensitivity is stronger below the peak temperature (<40 °C), while it weakens when the PCM is almost completely melted and is negligible after the peak temperature.

## 3. Summary and Comparison of the Investigated Composites

The case study presented in Section 2 demonstrates the lights and shades of multifunctional composites combining load-bearing and heat management functions. Despite the potential of such materials, the structural and TES properties are hardly ever synergistic: an increase in the MC weight fraction increases the TES capability and the total heat exchanged, but it decreases almost all mechanical properties.

This was also observed in other PCM-containing composites. Our group has recently investigated the concept of structural TES composites with a wide range of polymer matrices, reinforcements, and PCMs. The work encompassed (i) thermoplastic, thermosetting, and reactive thermoplastic matrices, (ii) traditional fibrous reinforcements constituted by the continuous or discontinuous glass and carbon fibers and less traditional reinforcements represented by thin beechwood laminae, and (iii) microencapsulated and shape-stabilized PCMs, added in variable weight fractions (up to ~30 wt%). All considered systems with references are listed in Table 3.

**Figure 6:**
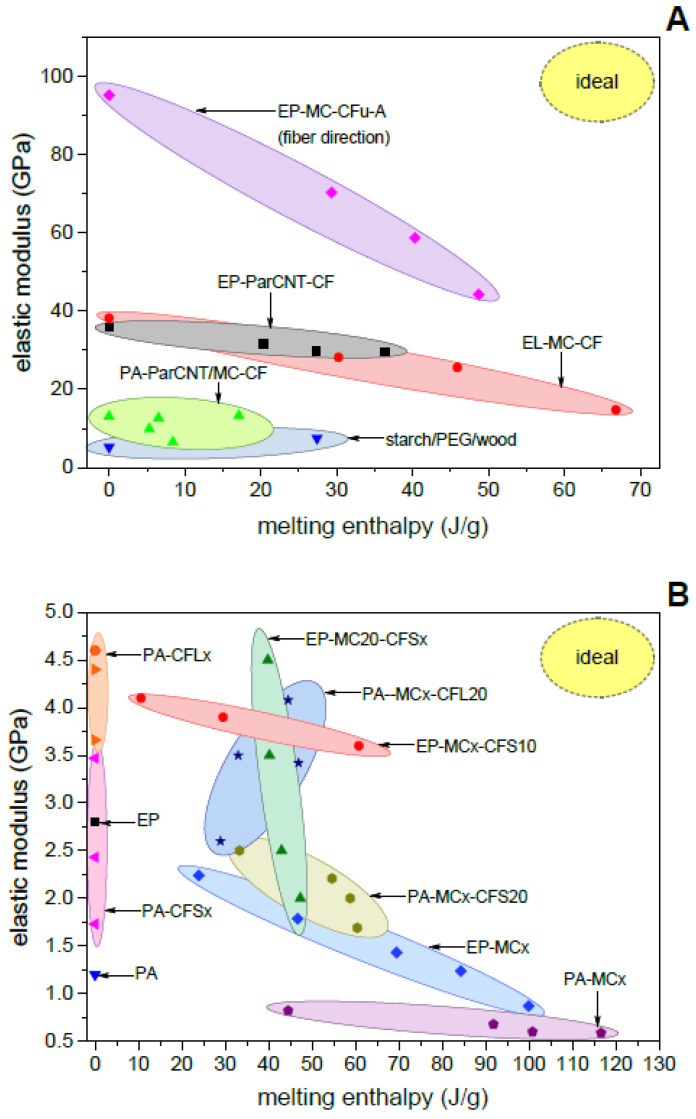
Relationship between elastic modulus and melting enthalpy of all evaluated composites (flexural modulus, except for the systems with a PA matrix and the starch/PEG/wood composites, for which the tensile modulus is reported). The ideal case is found in the top-right corner, with simultaneous maximization of stiffness and melting enthalpy. (**A**) Continuous-fiber composites. The plot reports values of modulus along the fiber axis for unidirectional composites and in the 0–90 direction for bidirectional composites.; (**B**) Discontinuous-fiber composites.

**Figure 7:**
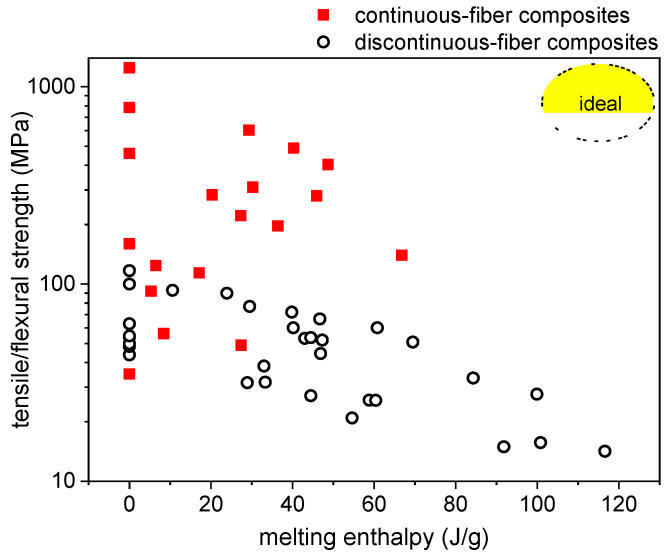
Relationship between mechanical strength (Log scale) and melting enthalpy of all evaluated composites (flexural strength, except for the systems with a PA matrix and the wood/starch/PEG composites, for which the tensile strength is reported). The ideal case is found in the top-right corner, with simultaneous maximization of mechanical strength and melting enthalpy.

To investigate the relationship between structural and TES performance and compare the properties of the different composites, the elastic modulus and the mechanical strength (structural parameters) of all composites can be plotted as a function of the melting enthalpy (TES parameter). The elastic modulus (Figure 6) generally decreases with increasing melting enthalpy. For continuous-fiber composites (Figure 6A), the decrease is mainly due to a decrease in the volume fraction of the reinforcement, as is also clear considering that the decrease is almost negligible when the fiber volume fraction has been forced constant by adjusting the processing parameters, such as in the laminates EP-ParCNT-CF [39]. Similar conclusions can be obtained from the results of discontinuous fiber composites Figure 6B), for which the fiber volume fraction is generally lower and easy to control and the modulus is significantly dependent also on the matrix stiffness. In this case, when the PCM fraction is constant (e.g., EP-MC20-CFSx), the melting enthalpy is constant and the modulus increases with the fiber content; when the fiber fraction is constant (e.g., EP-MCx-CFS10), the melting enthalpy increases and the elastic modulus slightly decreases with the PCM content. The same can be said for the mechanical strength (Figure 7), which generally decreases with increasing phase change enthalpy.

The ideal case would be a combination of high stiffness (and strength) and high melting enthalpy, which would be represented in the top right corner of Figure 6 and Figure 7. However, it is challenging to maximize both properties simultaneously, because the mechanical properties generally increase with the fiber volume fraction and the TES properties with the PCM weight fraction, while the experimental results show that fiber and PCM fractions generally follow opposite trends. The best materials for such composites would be a reinforcement with high specific (i.e., normalized to density) mechanical properties and a PCM with a large specific (i.e., normalized to mass) phase-change enthalpy so that the product between fiber volume fraction and PCM mass fraction would be maximized. In any case, the property to be maximized depends on the specific application, as well as the combination of properties to be considered optimal.

The trends Figure 6 and Figure 7 occur because, for most of the studied systems, the reinforcement does not contribute to store heat and the PCM does not raise the stiffness and strength. To make structural and TES properties truly synergistic and not parasitic, the multifunctionality should be shifted from the level of the composite to the level of the single phase. This was achieved in one of the investigated systems, i.e., the starch/wood/PEG laminate. In this composite, thin beechwood laminae were impregnated with PEG, interleaved with thermoplastic starch sheets, and compacted by hot pressing. Here, the beechwood laminae acted both as reinforcement and shape-stabilizing agent for PEG, and PEG not only played the role of the PCM, but also significantly increased the tensile, impact, and dynamic-mechanical properties of the composite.

## 4. Evaluation of the Multifunctional Performance

To reconcile the competing design requirements of the presented multifunctional structural TES composites, it is important to define objective parameters that seize all functions of the investigated material. The following analysis is made with an approach similar to that developed by O’Brien et al. [55] for multifunctional structural composite capacitors and used by other authors for analogous systems [25,56,57]. The objective here is to minimize the system mass for a unit presenting both structural and TES requirements.

A conventional system is made of two monofunctional units, which perform the structural and the TES function and have masses $m_{s}\;$and $m_{TES}$, respectively, has a total mass (M) given in Equation (1):

(1) $$M=m_{s}\;+\;m_{TES}$$

The TES unit has a phase change enthalpy per unit mass (ΔH), measured in J/g, and the structural unit has a specific (normalized by density) elastic modulus ($\bar{E}$). If the TES unit does not perform any load-bearing function and the structural unit does not participate in the thermal management function, then the whole system has a total melting enthalpy of ΔH and a total specific modulus of $\bar{E}$. Hence, ΔH and $\bar{E\;}$are the parameters describing the performance of the full system.

If the system is composed not of two monofunctional units but of a multifunctional material, this material can be a structural TES material with a mass $m_{mf}^{*}$. Therefore, the total mass of the multifunctional system ($M^{*}$) is now the mass of this structural TES material, or, as described by Equation (2),

(2) $$M^{*}=m_{mf}^{*}$$

This new system, with a specific enthalpy of $\Delta H_{mf}^{*}$ and specific elastic modulus of ${\bar{E}}_{mf}^{*}$, should maintain the same structural and TES performance as that made of two monofunctional units, in terms of total absorbed and released energy (in J) and total elastic modulus (in GPa). The two conditions are described by Equations (3) and (4):

(3) $$\Delta H\cdot m_{TES}=\Delta H_{mf}^{*}\cdot m_{mf}^{*}$$

(4) $$\bar{E}\cdot m_{s}={\bar{E}}_{mf}^{*}\cdot m_{mf}^{*}$$

if a unit volume is considered. Hence, an effective mass saving is verified if Equation (5) is met:

(5) $$M-M^{*}=\frac{{\bar{E}}_{mf}^{*}}{\bar{E}}\cdot m_{mf}^{*}+\frac{\Delta H_{mf}^{*}}{\Delta H}\cdot m_{mf}^{*}-m_{mf}^{*}=\left(\frac{{\bar{E}}_{mf}^{*}}{\bar{E}}+\frac{\Delta H_{mf}^{*}}{\Delta H}-1\right)\cdot m_{mf}^{*}>0.$$

Or, as expressed in Equation (6),

(6) $$\frac{{\bar{E}}_{mf}^{*}}{\bar{E}}+\frac{\Delta H_{mf}^{*}}{\Delta H}>1.$$

It is now possible to define a structural efficiency $\eta_{s}\;$and a TES efficiency $\eta_{TES}$ as described in Equations (7) and (8), as

(7) $$\eta_{s}=\frac{{\bar{E}}_{mf}^{*}}{\bar{E}},$$

(8) $$\eta_{TES}=\frac{\Delta H_{mf}^{*}}{\Delta H},$$

and a multifunctional efficiency $\eta_{mf}\;$as described in Equation (9), as

(9) $$\eta_{mf}=\eta_{s}+\eta_{TES}$$

Therefore, the multifunctional material allows an effective mass saving when $\eta_{mf}>1$. This requirement can be met even if $\eta_{s}$ and $\eta_{TES}$ are individually lower than 1, i.e., if the multifunctional material has specific elastic modulus and specific phase change enthalpy lower than those of the monofunctional structural unit and the monofunctional TES unit, respectively.

The presented analysis can be directly applied to the composites investigated by our group to assess if some of the prepared systems would allow an effective mass saving. The parameters $\eta_{s}$, $\eta_{TES}$ and $\eta_{mf}$ were calculated for the prepared composites by considering as monofunctional structural units the respective composite without PCM, i.e., the neat EP-CF composite, the neat EL-CF composite, etc., which present an $\eta_{s}$ equal to 1 and an $\eta_{TES}$ equal to zero. Moreover, the respective microencapsulated or shape-stabilized PCM was considered as the monofunctional TES unit, although in a true monofunctional TES unit the PCM would probably be somehow macro-encapsulated.

**Figure 8:**
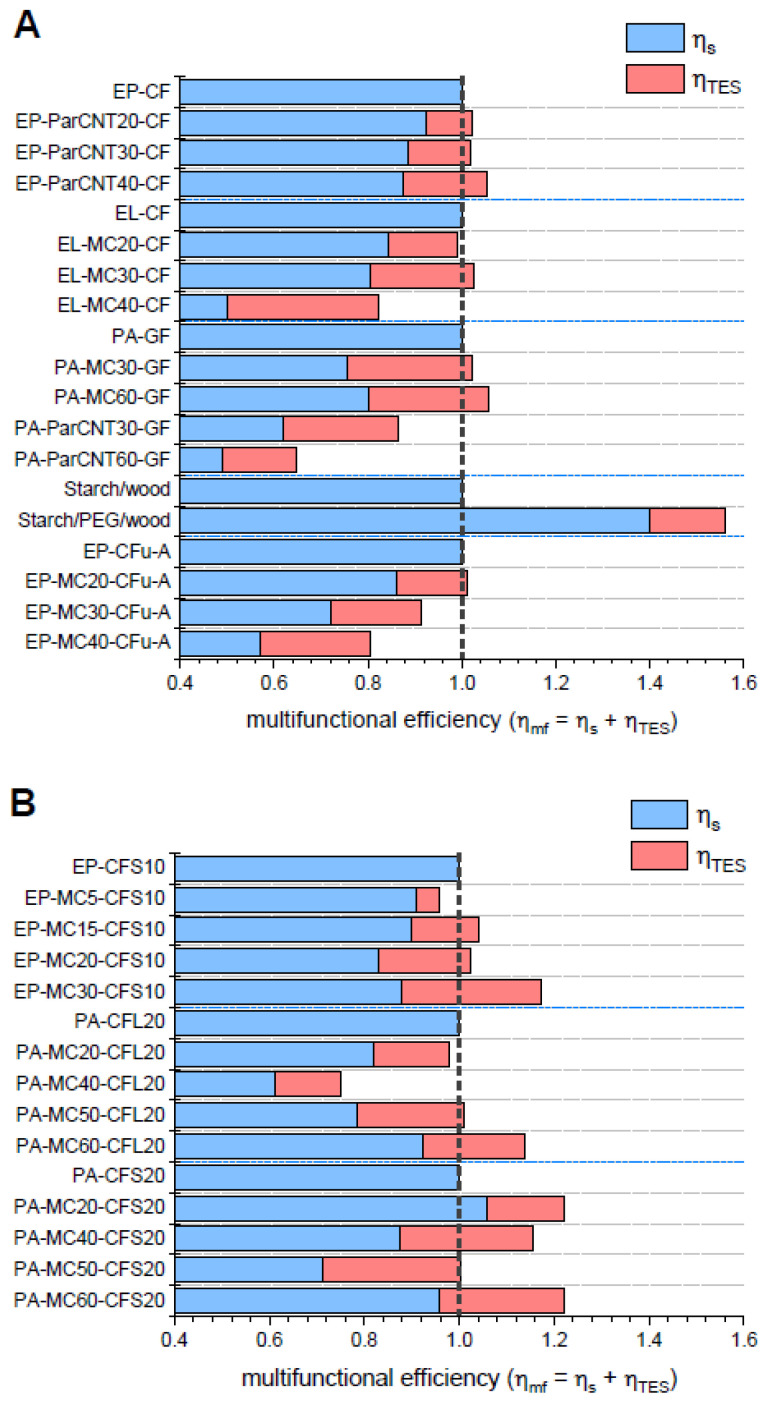
Multifunctional efficiency of the composites presented as the sum of structural and TES efficiencies. An effective mass saving is obtained when $\eta_{mf}$ is greater than 1. (**A**) Continuous-fiber composites; (**B**) discontinuous-fiber composites.

The data of $\eta_{s}$, $\eta_{TES}$ and $\eta_{mf}$ calculated with average data of elastic modulus, density, and phase change enthalpy, are presented in Figure 8A,B. For the composites containing continuous fibers (Figure 8A), a considerable mass saving is obtained for the system starch/wood/PEG, in which not only $\eta_{mf}$, but also $\eta_{s}\;$is greater than 1. This result is probably the consequence of the fact that the multifunctionality is at the phase level, as the wood laminae are both the reinforcement and the shape-stabilizing agent and PEG contributes to the mechanical properties of the laminae.

However, $\eta_{mf}$ is greater than 1 also for some other systems. This means that, even though the multifunctionality for these systems is not at the level of the single constituent but at the level of the whole composite material, they allow a certain (although lower) mass saving. For the composites EP-ParCNTx-CF and PA-MCx-GF, the multifunctional efficiency increases with the PCM content. This depends on two factors: (i) the fiber volume fraction was maintained constant, and (ii) the processing parameters were mild enough to avoid any critical modifications of most of the PCM. For the other systems, the maximum multifunctional efficiency is generally found at medium PCM concentrations, as for higher PCM concentrations not only does the PCM decrease the elastic modulus per se, but it also contributes to decreasing the fiber fraction.

For the composites containing discontinuous fibers (Figure 8B), for each system the pure monofunctional structural component was considered as that with the same matrix type and fiber fraction, but without PCM. In these systems, the fiber content is generally lower than that of continuous fibers, and the introduction of PCMs does not influence the fiber weight fraction, even though it could sometimes slightly decrease the total fiber volume fraction because the density of the used PCMs is generally slightly lower than that of the employed polymer matrices. For the systems EP-MCx-CFS10 and PA-MCx-CFS20, the multifunctional efficiency is generally higher than 1 and increases with the PCM content, again because the fiber content is nearly constant, and the processing conditions preserve most of the PCM from degradation.

This approach allows identifying the composition that maximizes the material’s multifunctional efficiency. However, this analysis is only valid if it considers parameters that are the most significant for a specific application, as for other cases it may be more meaningful to perform a volume-saving analysis or to maximize other properties such as the thermal conductivity, fracture toughness, or mechanical strength.

## 5. Conclusions and Future Perspectives

This work presented the concept of structural TES composite as a multifunctional material composed of a polymer matrix, a reinforcing agent, and a phase change material. This concept was introduced through a case study of epoxy/carbon laminates containing phase change microcapsules in variable weight fractions. In these laminates, as the MCs increased the matrix viscosity, the final matrix volume fraction increased with the MC concentration, with a consequent reduction of the fiber fraction. This phenomenon was on the basis of the poorer mechanical performance of the MC-richer laminates, although a small amount of MCs contributed to some mechanical properties such as the interlaminar fracture toughness. Moreover, the PCM phase is generally weak and not optimized to be used as a filler in a polymer matrix, which also contributes to decreasing the mechanical properties of the host laminate. On the other hand, a higher MC fraction increased the TES performance and the total heat exchanged.

This ambivalent role of the PCM, which raises the TES properties but hardly ever contributes to the mechanical performance, was also observed in nearly all the investigated polymer composites containing a PCM. Nevertheless, the combination of reinforcing agents and PCMs in a composite may still bring substantial mass and volume saving compared to two monofunctional units performing the structural and TES functions individually.

This advantage can be quantified by the approach proposed in this paper. Here, the multifunctional efficiency ($\eta_{mf}$) was calculated for all the investigated composites as the sum of the structural and the TES efficiency, where the structural efficiency is the ratio between the specific elastic modulus of the multifunctional composite and that of the monofunctional structural unit (the composite without PCM), and the TES efficiency is the ratio between the specific melting enthalpy of the multifunctional composite and that of the monofunctional TES unit (the PCM). An effective mass saving is obtained when $\eta_{mf}$ is greater than 1. The higher value of $\eta_{mf}$ (1.57) was found for the starch/wood/PEG laminate, as the wood laminae are both the reinforcement and the shape-stabilizing agent for the PCM, and the PCM also increases the mechanical stiffness. However, $\eta_{mf}$ is greater than 1 also in some other composites, which indicates that these structural TES composites would allow a certain mass saving.

To maximize $\eta_{mf}$, two routes are recommended, i.e., (i) shifting the multifunctionality from the level of the composite down to the level of the single-phase, as in the case of the starch/wood/PEG laminate, and (ii) optimizing the material design and selection. For the first route, it could be beneficial to find other reinforcing agents with a porous structure that can also act as shape-stabilizers for the PCMs, or it can be useful to optimize the properties of the PCM microcapsules, in terms of the shell’s mechanical stiffness and adhesion with the polymer matrix. For the material design and selection, the results showed that semi-structural composites reinforced with discontinuous fibers have generally higher $\eta_{mf}$, thus being a better option when maximizing the phase change enthalpy is more important than reaching very high mechanical properties. For continuous fiber composites, since the PCM tends to weaken and thicken the interlaminar region, an improved design of the stacking sequence could concentrate most of the PCM in the core layers and leave the outer layers richer in the reinforcing phase. A further extension of this concept may consider sandwich structures where all the PCM is concentrated in the core and the mechanical resistance is demanded to the outer skins, thereby shifting the multifunctionality from the material level up to the structure level.

## Figures and Tables

**Table 1:** Main results of DSC tests and volumetric composition of the prepared laminates. Data from [41].

Sample	$T_{m}\;(\text{°}C)$	$\Delta H_{m}\;(J/g)$	$T_{c}\;(\text{°}C)$	$\Delta H_{c}\;(J/g)$	$\omega_{MC}\;(\mathrm{wt}\%)$	$\vartheta_{v}\;(\mathrm{vol}\%)$	$\vartheta_{f}\;(\mathrm{vol}\%)$	$\vartheta_{MC}\;(\mathrm{vol}\%)$
MC	46.5	221.7	27.6	223.5	100.0	-	-	100.0
EP-CFu-A	-	-	-	-	0.0	2.3	62.5	0.0
EP-MC20-CFu-A	45.4	29.3	27.0	29.8	13.2	6.8	39.4	17.5
EP-MC30-CFu-A	47.8	40.3	25.5	40.2	18.2	5.1	38.2	24.1
EP-MC40-CFu-A	48.2	48.7	24.9	48.7	22.0	8.5	35.6	27.7
EP-CFu-B	-	-	-	-	0.0	1.2	58.8	0.0
EP-MC20-CFu-B	49.2	30.6	23.8	30.5	13.8	4.9	37.6	18.5
EP-MC30-CFu-B	51.0	42.0	21.9	41.9	19.0	3.7	35.4	25.1
EP-MC40-CFu-B	53.7	47.5	19.4	47.1	21.4	8.1	29.2	26.5

$T_{g}$ = glass transition temperature of the epoxy phase (°C); $T_{m}$ = melting temperature of the PCM (°C); $\Delta H_{m}$ = PCM melting enthalpy (J/g); $T_{c}$ = crystallization temperature of the PCM (°C); $\Delta H_{c}$ = PCM crystallization enthalpy (J/g); $\omega_{MC}$ = experimental capsule weight fraction calculated from the measured melting enthalpy (wt%); $\vartheta_{v}$ = porosity (vol%); $\vartheta_{f}\;$ = fiber volume fraction (vol%); $\vartheta_{MC}\;$ = MC volume fraction (vol%). - = not applicable.

**Table 2:** Mode Ι critical strain energy release rates for crack initiation ($G_{Ii}$ and steady-state propagation ($G_{Ic}$) for the laminates EP-MCx-CFu-B. Reprinted with permission from [41].

Sample	$G_{Ii}\;(\mathrm{MPa}\text{·}m^{1/2})$	$G_{Ic}\;(\mathrm{MPa}\text{·}m^{1/2})$
EP-CFu-B	0.15 ± 0.03	0.27 ± 0.01
EP-MC20-CFu-B	0.34 ± 0.02	0.40 ± 0.01
EP-MC30-CFu-B	0.27 ± 0.02	0.32 ± 0.01
EP-MC40-CFu-B	0.24 ± 0.02	0.30 ± 0.02

**Table 3:** Multifunctional composites under investigation in this paper with specifications of matrices, reinforcing agents, and PCMs.

Label	Matrix	Reinforcement	PCM	Reference
PA-ParCNT-GF	Polyamide 12	Bidirectional glass fibers	CNT-stabilized paraffin	[38,40]
PA-MC-GF	Polyamide 12	Bidirectional glass fibers	Paraffin microcapsules	[40]
EL-MC-CF	Reactive acrylic thermoplastic	Bidirectional carbon fibers	Paraffin microcapsules	[47,48]
Starch/PEG/wood	Thermoplastic starch	Thin beechwood laminae	Poly(ethylene glycol) 600	[50]
EP-ParCNT-CF	Epoxy	Bidirectional carbon fibers	CNT-stabilized paraffin	[38,39]
EP-MC-CFu-A	Epoxy	Unidirectional carbon fibers	Paraffin microcapsules	[41]
EP-MCx	Epoxy	-	Paraffin microcapsules	[54]
EP-MCx-CFSy	Epoxy	Milled carbon fibers	Paraffin microcapsules	[46]
PA-MCx	Polyamide 12	-	Paraffin microcapsules	[43]
PA-CFLx	Polyamide 12	Chopped carbon fibers	-	[43]
PA-CFSx	Polyamide 12	Milled carbon fibers	-	[43]
PA-MCx-CFLy	Polyamide 12	Chopped carbon fibers	Paraffin microcapsules	[43]
PA-MCx-CFSy	Polyamide 12	Milled carbon fibers	Paraffin microcapsules	[43]

## Data Availability

Data supporting the findings of this study are available on request by the corresponding author.
